# Ghrelin in Female and Male Reproduction

**DOI:** 10.1155/2010/158102

**Published:** 2010-03-14

**Authors:** Joëlle Dupont, Virginie Maillard, Stéphanie Coyral-Castel, Christelle Ramé, Pascal Froment

**Affiliations:** Unité de Physiologie de la Reproduction et des Comportements, INRA, UMR85, 37 380 Nouzilly, France

## Abstract

Ghrelin and one of its functional receptors, GHS-R1a (Growth Hormone Secretagogue Receptor 1a), were firstly studied about 15 years. Ghrelin is a multifunctional peptide hormone that affects several biological functions including food intake, glucose release, cell proliferation… Ghrelin and GHS-R1a are expressed in key cells of both male and female reproductive organs in several species including fishes, birds, and mammals suggesting a well-conserved signal through the evolution and a role in the control of fertility. Ghrelin could be a component of the complex series of nutrient sensors such as adipokines, and nuclear receptors, which regulate reproduction in function of the energy stores. The objective of this paper was to report the available information about the ghrelin system and its role at the level of the hypothalamic-pituitary-gonadal axis in both sexes.

## 1. Introduction

Ghrelin was initially discovered as a ligand for the growth hormone secretagogue receptor (GHS-R1a) [1], and the story of its discovery has been well described in some reviews [2–4]. The peptide named “ghrelin” is a term derived from the Proto-Indo-European word “ghre” meaning “grow” and the name can also indicate the abbreviation for GH, followed by “relin” a suffix meaning releasing substance. Ghrelin is a peptide hormone secreted mainly by the stomach, although its expression has been detected in many other organs exerting both endocrine and paracrine effects [5–7]. Ghrelin has initially been reported to induce GH secretion [1]. In addition to mediating GH release through the growth hormone secretagogue receptor (GHS-R), ghrelin is involved in a series of biological functions including regulation of food intake [8], sleep [9], body weight [10], gastrointestinal motility [11], cardiovascular functions [12], cell proliferation [13], production of proinflammatory cytokines [14], and reproduction in many species. The objective of this paper is to review the available information on the role of ghrelin in reproductive processes including female and male reproduction.

## 2. Structure and Distribution of Ghrelin

### 2.1. Structure of Ghrelin

Ghrelin is a 28-amino acid peptide derived from preproghrelin [1]. It has two major endogenous forms: a des-acylated form (des-acyl ghrelin) and a form acylated at serine 3 (ghrelin). This posttranslational acylation is essential for the hormone biological activity [1, 4, 15]. The ghrelin structure, particularly that of the acyl-modification regions, is highly conserved throughout vertebrate species [1].

### 2.2. Distribution of Ghrelin

Ghrelin is found in mammalian species as well as nonmammalian species. The majority of ghrelin is synthesized by an endocrine cell population, the X/A-like cells, in the stomach mucosa [16]. Ghrelin is then released to the general circulation. The des-octanoylated ghrelin and n-octanoylated ghrelin are both found in rat stomach [17]. The small intestine also synthesizes ghrelin to a lesser extent with the amount of ghrelin produced diminishing with increasing distance from the pylorus [16, 18]. The expression of ghrelin has also been reported in pancreas, lymphocytes, placenta, kidney, lung, heart, pituitary, brain, ovary, and testis [5–7]. Thus, ghrelin is a ubiquitous protein.

### 2.3. Regulation of Ghrelin Expression and Secretion

Circulating ghrelin levels increase with fasting and return to basal levels after refeeding in rodents and humans [19–25]. The plasma ghrelin concentration in cows decreases significantly one hour after feeding, and then recovers to prefeeding levels [26]. Starvation also increases plasma ghrelin level in prepubertal gilts [27]. Nutrient contents and also hormones are important factors for the regulation of ghrelin expression and release. For example, in the female rat stomach, estrogen decreases ghrelin mRNA expression [28]. In contrast, the ghrelin mRNA level in the rat stomach increases after the administration of insulin and leptin [17]. In cultured whole porcine follicles, GH stimulates both ghrelin synthesis and secretion, whereas IGF-I shows less influence [29]. In rodents, growth hormone-releasing hormone upregulates ghrelin mRNA in the pituitary [30].

## 3. Ghrelin Receptor

### 3.1. Structure

The ghrelin receptor is a G-protein-coupled receptor (GPCR) firstly identified in pigs and humans in 1996 [31]. It belongs to the rhodopsin-like seven-transmembrane domain (7TM) receptor family that includes the orphan GPR39 as well as receptors for the peptides motilin, neurotensin, and neuromedin [32]. It has a high degree of homology ranging from 93% to 99% identified by using molecular analysis of human, pig, dog, rat, and mouse species [31–35]. Cloned before the discovery of the peptide, the ghrelin receptor was initially described as the receptor for a number of synthetic GH secretagogues and is therefore also called the growth hormone secretagogue receptor (GHS-R1a) [31, 36]. However, some evidence indicates that several ghrelin effects are mediated not by GHS-R1a but by other types of receptors not yet identified [37]. An inactive alternative splice variant of the GHS-R subtype, termed GHS-R1b, has also been found [31]. Unlike GHS-R1a, GHS-R1b is not activated by the synthetic GHSs or ghrelin and it is unclear whether it is a functional receptor [38].

### 3.2. Distribution

The GHS-R1a receptor mRNA is mainly expressed in the pituitary [31] and in several structures of the brain of mammalian and nonmammalian species [39–42]. However, it is also present in the thyroid, pancreas, spleen, myocardium, adrenal gland, ovary, and testis [43]. These data suggest direct actions of ghrelin in these tissues.

### 3.3. Signaling Pathways

Ghrelin endocrine activities depend entirely upon the acylation and are mediated by GHSR-1a. The des-acyl ghrelin does not bind to GHSR-1a. Upon ghrelin binding to its receptor, different negative feedbacks of GHSR-1a have been described. After acute treatment of porcine pituitary cell cultures with ghrelin, Luque et al. found that ghrelin downregulated GHS-R expression [44]. Ghrelin binding to GHS-R1a also results in a rapid attenuation of the receptor responsiveness. This desensitization is the result of the uncoupling of the receptor from heterotrimeric G proteins and of the internalization of the cell surface receptors to intracellular compartments [45, 46]. Ghrelin is able to activate various signaling pathways. In GHS-R1a-expressing mammalian cells [1, 31] or in rat and human pituitary cells [47, 48], biphasic Ca^2+^ increases, due to a transient Ca^2+^ release from the intracellular store and a Ca^2+^ influx through voltage-dependent L-type calcium channel, which are observed as the signal transduction. Ghrelin has also been shown to increase AMPK (Adenosine Monophosphate-activated kinase) activity in the hypothalamus [49] and reduce it in the liver [50, 51]. It has also been reported that ghrelin could activate the MAPK 44- and 42-kDa extracellular signal-regulated protein kinases (ERK1/2) and the Akt [43–50] in different cell lines [52–55]. Ghrelin is also able to regulate the expression of several transcription factors including the nuclear factor *κ*B, (NF*κ*B), PPAR*γ*, SREBP-1, and cEBP [56, 57].

## 4. Physiological Functions of Ghrelin in Reproductive Tissues

About ten years ago, several experiments suggested that ghrelin could act as a modulator of the male and female reproductive functions. Indeed, many in vivo and in vitro studies showed that ghrelin was able to exert its action at different levels of the hypothalamic-pituitary-gonadal axis.

### 4.1. Ghrelin and Gonadotropin-Releasing Hormone Secretion (GnRH)

The hypothalamus has been identified as the main source of ghrelin in the central nervous system. Furthermore, as previously described, the GHS-R1a receptor mRNA has been found in many areas of the brain. In rats, systemic administration of ghrelin reduces in vivo the GnRH pulse frequency. The involvement of NPY in the mediation of the effects of ghrelin on pulsatile GnRH secretion is indicated by the complete abolition of the effects of ghrelin by the NPY-Y5 receptor antagonist [58]. GnRH secretion by hypothalamic fragments from ovariectomized females is also significantly inhibited by ghrelin [59]. In mammalian and nonmammalian species, ghrelin affects gonadotropin release acting at the level of the hypothalamus as well as directly on the pituitary gland [60].

### 4.2. Ghrelin and Gonadotropin Secretion

In pituitary, ghrelin suppresses LH pulse frequency in rats, sheep, monkeys [61–63], and humans [64]. Furthermore, ghrelin delays pubertal onset in male rats [59]. In rats, ghrelin is able to downregulate *Kiss1* expression in the hypothalamic medial preoptic area and this could be a contributing factor in ghrelin-related suppression of pulsatile LH secretion [65]. In contrast, in women during the menstrual cycle, administration of ghrelin does not affect basal and GnRH-induced LH and FSH secretion [66]. Opposite effects of ghrelin on LH secretion mammals and several fish species have been described. Indeed, ghrelin has been shown to stimulate LH release in goldfishes [67–69] and recently in carps [70]. Recent studies indicate that synthetic goldfish ghrelin stimulates LH release [71]. However, the specific mechanism and the role of this activation are still unknown.

There is evidence in rats and humans that ghrelin can suppress not only LH but also FSH secretion in males and females [63]. A significant decrease of FSH was observed after seven days of continuous ghrelin infusion in male rats [64] and in the metestrus of female rats after one injection. However, in rats, ghrelin did not affect FSH secretion in the proestrous and estrous periods of the estrous cycle in females, and in gonadectomized male and female rats after single injection [64] and after chronic intermittent administration [64]. In women during the menstrual cycle, administration of ghrelin did not affect basal and GnRH-induced LH and FSH secretion [66].

The reported effects of ghrelin on LH and FSH secretion suggest that this peptide plays a key role in the reproductive functions. Beside central actions on the reproductive functions, some evidence indicates that ghrelin could exert direct effects on the female and male gonads.

### 4.3. Ghrelin in Female Reproduction

Emerging evidence strongly indicates that the ghrelin and ghrelin receptor (GHS-R1a and GHS-R1b) are present in the mammalian and nonmammalian ovary. For example, ghrelin is found in human, rat, pig, sheep, and chicken ovary [72–76]. In sheep ovary, ghrelin is expressed throughout the estrous cycle and pregnancy and the relative mRNA levels depend on the stage of the cycle, with the highest expression during the development of the corpora lutea (CL) and minimal expression in the regressing CL. A similar pattern is seen during pregnancy [77]. More precisely, in rodent ovary, expression of ghrelin has been demonstrated in steroidogenically active luteal and interstitial hilus cells. Expression of the functional ghrelin receptor has been reported in oocytes as well as follicular, luteal, and surface epithelium and interstitial hilus cells in rat ovary [72, 75, 78]. These observations indicate that ovarian follicular and luteal cells are potential targets for systemic or locally produced ghrelin, because they express the functional type 1a of GHS-R. They also highlight the plausibility for a role of ghrelin in the direct control of ovarian cell functions. In vivo administration of ghrelin in rats affects folliculogenesis as attested by alterations of some morphometrical and intracellular indexes in ovarian state. Indeed, it decreases the mean diameter of follicles, the number of corpora lutea, luteal cells, and oocyte and the diameter of the theca layer and the zona pellucida as well as the whole ovarian volume in the treated animals [79].

#### 4.3.1. Effect of Ghrelin on Ovarian Steroidogenesis (Figure 1)

In cultured human granulosa luteal cells, ghrelin exerts an inhibitory effect on steroidogenesis (progesterone and estradiol production) in the absence or in the presence of hCG by acting through its functional GHS-R1a [80, 81]. Moreover, the granulosa cells from ghrelin-treated rabbits secrete not only less progesterone and estradiol but also less IGF-1 and prostaglandin F than granulosa cells from untreated animals [82]. In contrast to previous reports, in cocultured granulosa and theca cells from porcine follicles, ghrelin induced estradiol secretion by modifying aromatase activity [29, 37]. Also, Ghrelin(1–18) administration in chicken causes not only an increase in progesterone and oestradiol but also secretion of arginine vasotocin and IGF-1 [76, 83].

#### 4.3.2. Effect of Ghrelin on Ovarian Cell Proliferation and Apoptosis (Figure 1)

In chicken ovarian cells, in vitro ghrelin treatments induce markers of proliferation [MAP kinase; PCNA (proliferating cell nuclear antigen), a marker of the S/phase of the cell cycle, and cyclin B1, a marker of the G2/phase] and decrease the expression of markers of apoptosis (caspase-3, bax, and bcl-2) [76, 84]. Moreover, granulosa cells from ghrelin-treated rabbits have higher expression of PCNA and lower expression of TdT (terminal deoxynucleotidyl transferase), than those from control animals. 

#### 4.3.3. Effect of Ghrelin on Oocyte Maturation and Embryo Development (Figure 1)

It has also been reported that ghrelin inhibits early embryo development in mice [85]. In porcine oocytes cultured in vitro, ghrelin does not improve meiotic maturation. In contrast, it may have some inhibitory effects on the organization of microtubules and microfilaments of porcine oocytes [86]. On the contrary, some data suggest that ghrelin could enhance blastocyst viable from porcine oocytes fertilized in vitro and parthenogenetic embryos while exerting a negative effect on the structural integrity of the blastocysts [87]. Thus, the effects of ghrelin on the development of the embryo are not clear. 

In addition, in rats, high levels of ghrelin receptor (GHS-R) mRNA are detected in various peripheral fetal tissues beginning on embryonic day 14 and lasting until birth. Maternal ghrelin regulates fetal development during the late stages of pregnancy [88].

### 4.4. Ghrelin in Male Reproduction

**Figure 1:**
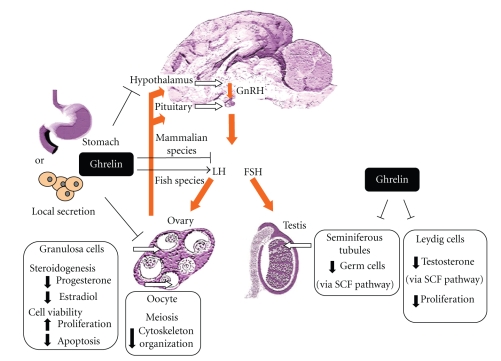
Schematic representation of the ghrelin effects at the level of the hypothalamic-pituitary-gonadal axis. Ghrelin, mainly produced by the stomach, can act through its functional receptor GHS-R1a in endocrine or/and local manner in all male and female reproductive tissues including hypothalamus, pituitary, ovary, and testis. It is well known that ovarian steroid production (oestradiol and progesterone) can modulate pituitary and hypothalamus secretions. Furthermore, GnRH produced by the hypothalamus controls LH, and FSH secretion that is known to regulate gonad functions. In mammalian species, ghrelin treatment inhibits GnRH, LH and FSH secretion at the hypothalamic and pituitary levels (red arrows). Opposite effects have been described in several species of fish. In the gonads, ghrelin exerts also inhibitory effects by altering steroidogenesis and germ cells production or viability in ovary and testis. In contrast, ghrelin treatment reduces proliferation of Leydig cells whereas it increases those of granulosa cells. SCF pathway: Stem Cell Factor pathway. ↓: decrease, ↑: increase, and ⊥: inhibition.

The testis is a complex endocrine organ where different cell types interplay to produce germ cells, under the control of several extragonadal and intragonodal hormones and growth factors. Some evidence suggests that ghrelin participates in such a regulatory network [59, 89–91] (Figure 1).

Expression of ghrelin has been demonstrated in rodents and sheep by immunostaining mainly in Leydig cells [91]. Ghrelin is also present in the human testis and particularly in Leydig and Sertoli cells but not in germ cells [92]. In human testis, the expression of ghrelin by Leydig cells is apparently linked to the degree of cell differentiation [92]. Furthermore, it is inversely correlated with the serum testosterone levels in patients with normozoospermia, obstructive azoospermia, or varicocele suggesting that ghrelin has an indirect effect on spermatogenesis [93]. In contrast to human and rodent data, in adult sheep testis, strong ghrelin immunostaining is evident not only in Leydig and Sertoli cells but also in germ cells, with an indication of increased ghrelin immunoreactivity in germ cells during the mitotic phases and the meiotic prophases of the spermatogenic cycle [74]. Thus, there are some differences between species in the localization of ghrelin protein in the testis. 

Expression of the functional ghrelin receptor, GHS-R1a, has been shown in Sertoli and Leydig cells as well as seminiferous tubules in rats [79]. Some changes in the balance between 1a and 1b isoforms of GHS-R gene have been described in rat testis. Indeed, changes in the alternative splicing of the gene are observed throughout postnatal development [94]. Specifically, during pubertal development, a shift in the pattern of splicing of GHS-R gene takes place in rat testis, favouring the expression of the biological active type 1a form of the receptor and indicating that the balance between receptor subtypes may represent a novel mechanism for the regulation of ghrelin sensitivity in gonads. In humans, GHS-R1a has been located in germ cells, mainly in pachytene spermatocytes, as well as in Leydig and Sertoli cells [92]. In adult sheep, GHSR-1a protein was detected in Leydig cells as well as in Sertoli and germ cells within the tubules, and the pattern of GHSR-1a mRNA expression across the testis indicated that the mRNA was present in the interstitial area and around the periphery of the tubules.

#### 4.4.1. Effect of Ghrelin on the Seminiferous Tubule Functions (Figure 1)

These latter data suggest that ghrelin could regulate spermatogenesis by an autocrine or/and a paracrine manner. In this sense, intratesticular injection of ghrelin (15 *μ*g for 2 days) in adult rats inhibited mRNA expression of the gene encoding stem cell factor (SCF), a key signal for germ cells production and a putative regulator of Leydig cell development. Such an inhibitory action of ghrelin on SCF has also been detected in vitro using cultures of staged seminiferous tubules [5]. The testicular SCF is a Sertoli cell product that has been involved in Leydig cell development and survival and is acting as a survival factor for the different cell types in the seminiferous epithelium such as spermatogonia in adult rats [95]. Thus, the actions of ghrelin on tubular SCF mRNA could have an impact on the regulation of spermatogenesis and also on Leydig cell proliferation.

#### 4.4.2. Effect of Ghrelin on Testicular Steroidogenesis (Figure 1)

In vitro, ghrelin significantly also inhibits in a dose-dependent manner both hCG- and cAMP-stimulated testosterone release by Leydig cells [91]. This inhibitory effect of ghrelin on testosterone secretion has been associated with decreases in the hCG-stimulated expression levels of the mRNAs for several key factors in the steroidogenic pathway (StAR, P450scc, 3ß-HSD, and testis-specific 17*β*-HSD type III) [91]. In vivo, the effects of ghrelin on plasma levels of testosterone in rats depend on the nutritional state. Indeed, in fed rats, ghrelin administration induces a slight decrease in testis mass without detectable changes in final plasma levels of testosterone, whereas in food-restricted animals, where endogenous ghrelin levels are known to be increased, a chronic administration of ghrelin induces overt decrease in plasma testosterone [96]. Thus, high levels of ghrelin could contribute to male reproductive axis alterations in situations of energy deficit.

#### 4.4.3. Effect of Ghrelin on Cell Proliferation (Figure 1)

 It has also been demonstrated by in vivo intratesticular injection of ghrelin that ghrelin is able to inhibit the proliferative rate of immature Leydig cells both during puberty development and after selective ablation of pre-existing mature Leydig cells by administration of EDS (ethylene dimethane sulfonate) [5]. Ghrelin and its type 1a GHS-R are expressed in testicular tumors. The expression of ghrelin but not that of GHS-R1a in Leydig tumor cells is apparently linked to the degree of cell differentiation [92]. 

Ghrelin is able to modulate key testicular functions such as seminiferous tubule gene expression, testosterone secretion, and Leydig cell proliferation. Thus, this peptide could operate as a novel regulator of testicular development.

## 5. Conclusions

In conclusion, ghrelin is a peptide hormone mainly secreted from the stomach into the circulation, but it can be synthesized by other tissues such as reproductive tissues suggesting local actions (autocrine and/or paracrine). Its functional receptor, GHS-R1a, is also expressed at different levels of the hypothalamic-pituitary-gonadal axis. As described in this paper, ghrelin may participate in the regulation of different aspects of the female and male reproductive functions from germ cell production to embryo development. These actions appear to be species-specific. Ghrelin through its various biological functions including energy metabolism by promoting fat deposition and food intake could be a key signal between energy status and control of fertility (nutrient-gene expression). However, further studies are required to gain insights into the understanding of the fine mechanisms of ghrelin action.
